# Balloon-Assisted Thrombin Injection in Iatrogenic Common Femoral Artery Pseudoaneurysms With Long Wide Necks: Two Case Reports

**DOI:** 10.7759/cureus.81383

**Published:** 2025-03-28

**Authors:** Srijit Saha, Sounak Paul, Avik Bhattacharyya, Sourav Tripathy, Anasua Chattopadhyay

**Affiliations:** 1 Interventional Radiology, CK Birla Hospitals, The Calcutta Medical Research Institute, Kolkata, IND; 2 Radiodiagnosis, CK Birla Hospitals, The Calcutta Medical Research Institute, Kolkata, IND

**Keywords:** balloon-assisted thrombin injection, case report, femoral artery pseudoaneurysm, iatrogenic pseudoaneurysm, ultrasound-guided thrombin injection

## Abstract

Common femoral artery pseudoaneurysms can develop following traumatic events or as a complication of endovascular interventional procedures. There is a wide range of treatment options.

We present two cases, where the patients had developed pseudoaneurysms in the right common femoral artery following conventional coronary angiography. The aneurysms had long and wide necks and were managed successfully with balloon-assisted thrombin injection.

This is a novel technique that can prevent distal embolism and is mentioned in the literature. This is a potential alternative to surgical or other commonly performed procedures for a selected group of patients.

## Introduction

Common femoral artery (CFA) pseudoaneurysms (PSA) can develop following traumatic events or as a complication of endovascular interventional procedures [1]. There is a considerable risk of morbidity and mortality [2]. 

Some of the smaller pseudoaneurysms spontaneously thrombose with time. Other treatment options are direct compression under ultrasound guidance and injection of thrombin directly into the sac. Endovascularly, this can be treated with coils or covered stents. Open surgical repair is reserved for pseudoaneurysms with unfavorable anatomy [3].

Thrombin injection under ultrasound guidance has been reported as safe and effective treatment for peripheral artery pseudoaneurysms but there is risk of distal thromboembolism [4]. 

Balloon-assisted thrombin injection (BATI) is a relatively new technique, which has been reported in a few studies with favorable results [5,6].

We have described two cases where CFA pseudoaneurysms were treated with BATI to avoid distal embolism.

## Case presentation

Both patients were elderly females, ages 71 and 58 years. They had high body mass index (BMI) and presented with exertional angina. They underwent conventional coronary angiography via transfemoral route, as bilateral radial accesses were technically infeasible due to small radial arteries (<2mm in size) which went into spasm during initial radial access. Haemostasis was attempted by manual compression.

**Figure 1:**
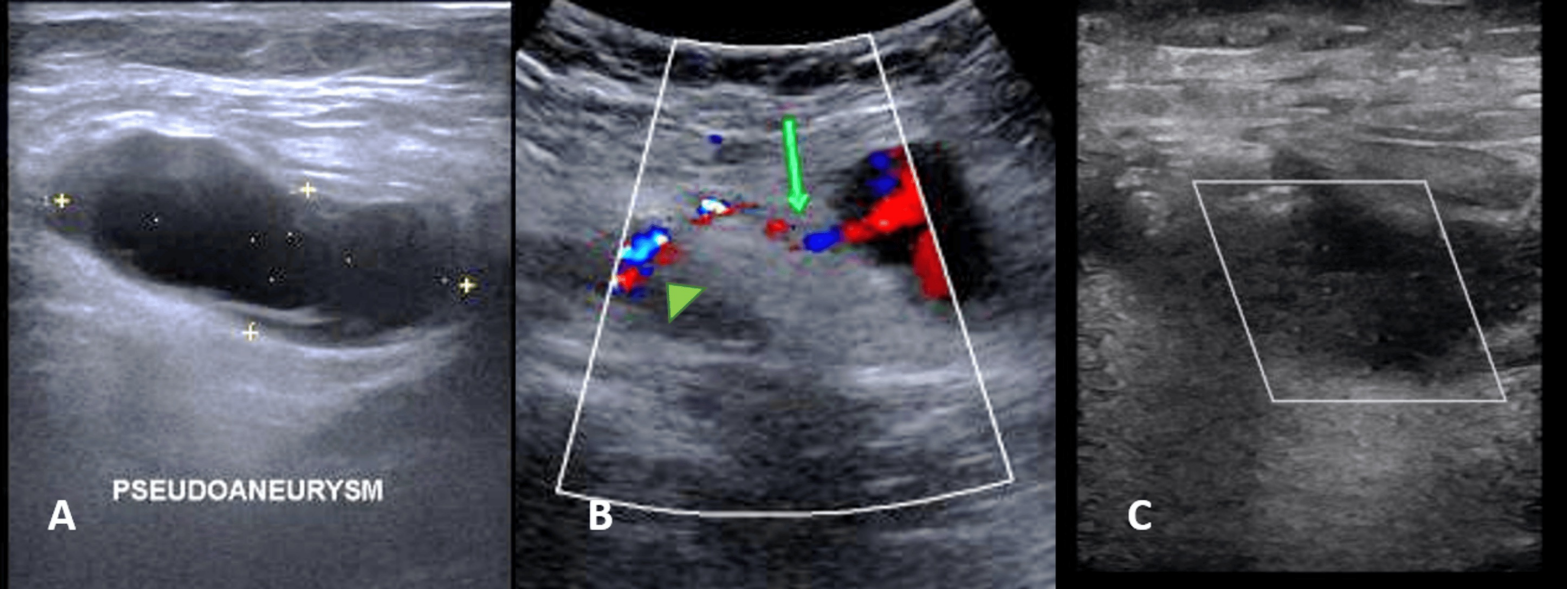
First patient, Ultrasound image of right groin A- Grey scale ultrasound shows an anechoic oval lesion with surrounding hematoma. B- Colour Doppler suggestive of a pseudoaneurysm with long wide neck (green arrow head at arterial wall with wide neck, green arrow at neck adjacent to pseudoaneurysm) arising from the common femoral artery (CFA). C- Post thrombin injection, ultrasound shows complete thrombosis of the sac and the neck.

The next day, the first patient developed a painful lump in the right groin. On ultrasound, a 42 x 16 mm PSA with a long wide neck (length 20 mm, width 4.3 mm) communicating with the right common femoral artery was seen. The sac was surrounded by hematoma (50 x 34 mm) and diffuse soft tissue edema (Figure 1A, 1B).

**Figure 2:**
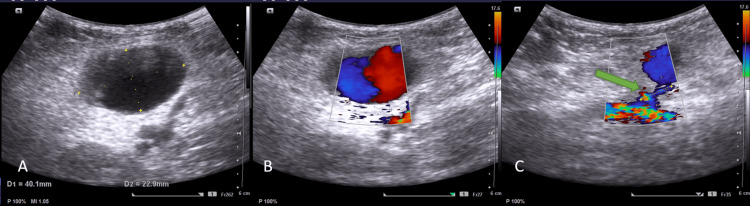
Second patient, Pre-procedure ultrasound images of right groin A- Grey scale ultrasound shows an anechoic oval lesion. B, C- Colour Doppler suggestive of a pseudoaneurysm (B) with long wide neck (green arrow) arising from the common femoral artery (CFA) (C).

The second patient developed a hard lump over the right groin with a bruise on the following day. On ultrasound, there was a pseudoaneurysm (40 x 22.9 mm) arising from the right CFA with a long wide neck (length 15 mm and width 3.7 mm) (Figure 2A-2C).

**Figure 3:**
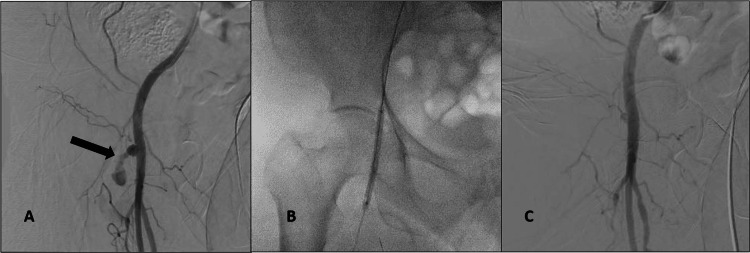
First patient, Digital Subtraction Angiography A- Common femoral artery (CFA) angiography shows wide neck pseudoaneurysm (PSA) (black arrow) from the right CFA. B- A 6 mm angioplasty balloon across the neck of the pseudoaneurysm. C- Post procedure, maintained flow in the right CFA, superficial femoral artery (SFA) with complete occlusion of the pseudoaneurysmal sac.

**Figure 4:**
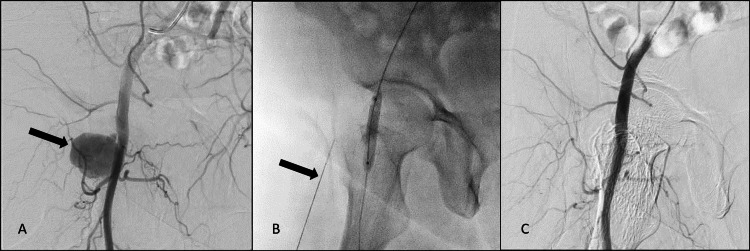
Second patient, Digital Subtraction Angiography A- Common femoral artery (CFA) angiography shows wide neck pseudoaneurysm (PSA) (black arrow) from the right CFA. B- A 7 mm angioplasty balloon was inflated across the neck of the pseudoaneurysm. A 22Gz needle (black arrow) is placed within the pseudoaneurysm sac. C- Post procedure, there is maintained flow in the right CFA with non-visualisation of the sac.

Both were planned for embolization of the pseudoaneurysmal sac, but the wide neck of the PSA posed a challenge. 5 Fr angiosheaths were inserted into the left common femoral arteries, and angiograms of the right external iliac artery revealed the wide neck PSAs from the right CFA. Subsequently, 6 mm and 7 mm angioplasty balloons were used in patient 1 and patient 2 respectively to occlude the neck of the pseudoaneurysms followed by injection of 3 ml reconstituted thrombin (Tisseel kit; Baxter, Deerfield, IL, USA, as per the product usage guidelines) into the sacs. Post-procedure, there was maintained flow in the right superficial femoral artery (SFA) with complete occlusion of the pseudoaneurysmal sacs (Figures 3A-3C, 4A-4C).

**Figure 5:**
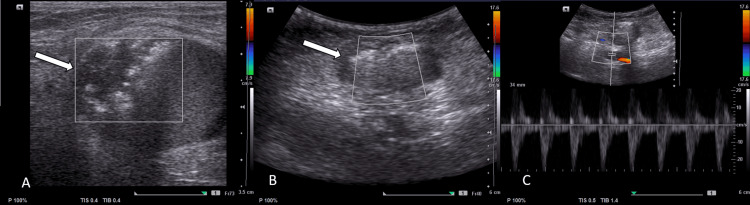
Second patient, Post-procedure ultrasound images of right groin A, B- Post thrombin injection, ultrasound shows complete thrombosis of the sac (white arrow). C- There is maintained flow in the right common femoral artery (CFA).

Review ultrasound on the next day showed complete thrombosis of the sacs with maintained flow in the right CFA, SFA, and distal arteries (Figures 1C, 5A-5C).

## Discussion

The cases describe the technical efficacy of BATI for a complicated PSA with a wide neck. Ultrasound-guided compression is a non-invasive but painful and time-consuming alternative with a high recurrence rate and is difficult in obese patients [7]. Injecting thrombin into a pseudoaneurysm with a wide neck is challenging due to the risk of local thrombosis and distal embolism leading to limb ischemia [8,9]. Covered stents need long-term anticoagulant therapy and are not suitable across the joint.

In our cases, occlusive balloon was deployed to protect the native CFA. The balloon inflation was done at low pressure (1-2 atm, just enough to occlude rent in the wall), to minimize intimal injury to the parent vessel. The balloon was deflated two to three minutes after thrombosis of the PSA was achieved. This prevented distal embolization and local thrombosis of the CFA.

There are a few published cases of balloon-assisted thrombin injection [5,6]. This technique has also been used in carotid and subclavian arterial PSAs [10,11].

The risk of local thrombosis of the parent artery or distal embolism in PSAs with complex anatomy is reduced with BATI. Further, the PSA sac and circulation in the arteries can be assessed during the procedure.

Open surgery risks, such as general anaesthesia and groin wound complications, can be avoided.

However, BATI comes with a few limitations. There lies a risk of arterial dissection due to prolonged inflation of the angioplasty balloon. Complications may arise in the contralateral site of access [12]. These can be avoided by low-pressure balloon inflation and adequate compression in contralateral groin or by use of closure devices.

## Conclusions

We presented two patients who had developed right common femoral artery PSA after a diagnostic endovascular procedure. Due to the complex anatomy of the PSA neck, balloon-assisted thrombin injection was done, which yielded successful results. It is a potential alternative to surgical or other commonly performed procedures for a selected group of patients as described previously.
